# The Efficacy and Safety Associated with Switching from Concomitant Brimonidine and Ripasudil, or Brimonidine or Ripasudil Monotherapy to a Fixed Combination of Brimonidine and Ripasudil in Glaucoma Patients

**DOI:** 10.3390/jcm13144158

**Published:** 2024-07-16

**Authors:** Hiromitsu Onoe, Kazuyuki Hirooka, Tetsuya Baba, Mikio Nagayama, Atsushi Hirota, Katsuyoshi Suzuki, Takeshi Sagara, Hideki Mochizuki, Yoshiaki Kiuchi

**Affiliations:** 1 Department of Ophthalmology and Visual Science, Hiroshima University, Hiroshima 734-8551, Japan; onoehir@hiroshima-u.ac.jp (H.O.); ykiuchi@hiroshima-u.ac.jp (Y.K.); 2 Shirai Eye Hospital, Mitoyo 767-0001, Japan; tbaba@shirai-hosp.or.jp; 3 Nagayama Eye Clinic, Okayama 714-0086, Japan; mikio@po.harenet.ne.jp; 4 Hirota Eye Clinic, Yamaguchi 745-0017, Japan; hirotaganka@lion.ocn.ne.jp; 5 Suzuki Eye Clinic, Yamaguchi 755-0155, Japan; suzuki_eye@grace.ocn.ne.jp; 6 Sagara Eye Clinic, Yamaguchi 758-0021, Japan; tsagara65@gmail.com; 7 Kusatsu Eye Clinic, Hiroshima 733-0861, Japan; mochizuki-h@hiroshima-u.ac.jp

**Keywords:** brimonidine, ripasudil, glaucoma

## Abstract

**Objectives**: The purpose of this study was to investigate switching from brimonidine and ripasudil, and brimonidine or ripasudil, to a fixed combination of brimonidine and ripasudil, and evaluate the associated efficacy and safety in glaucoma patients. **Methods**: Glaucoma patients undergoing treatment with at least brimonidine and ripasudil (n = 25) or treatment with at least brimonidine or ripasudil (n = 45) were evaluated in this retrospective study. After switching patients taking brimonidine and ripasudil, or brimonidine or ripasudil, to a ripasudil/brimonidine fixed-combination, ophthalmic suspension (RBFC), intra-ocular pressure (IOP), conjunctival hyperemia and superficial punctate keratopathy (SPK) were evaluated before and at 4, 12 and 24 weeks after switching to RBFC. **Results**: No significant differences in the IOPs were observed after switching from brimonidine and ripasudil to RBFC. However, a significant decrease was observed at 4, 12 and 24 weeks in the baseline IOP, from 17.0 ± 4.4 mmHg to 15.7 ± 3.2 mmHg (*p* < 0.01), 14.3 ± 3.4 mmHg (*p* < 0.01) and 14.4 ± 4.1 mmHg (*p* < 0.01), respectively, after switching from brimonidine or ripasudil to RBFC. No significant changes were noted for the SPK score or conjunctival hyperemia score at any of the visits after switching to RBFC. **Conclusions**: Throughout the 24-week evaluation period, the IOP was maintained after switching from brimonidine and ripasudil to RBFC. However, there was a significant decrease in the IOP after switching from brimonidine or ripasudil to RBFC. These results demonstrate that RBFC is safe for use in the treatment of glaucoma patients.

## 1. Introduction

The most common cause of irreversible blindness in the world is glaucoma [1], with an elevated intra-ocular pressure (IOP) the most important risk factor for developing the condition. It has been reported that lowering the IOP can decrease the speed of glaucoma progression [2]. In order to reduce the IOP, the goal of treatment strategies is to decrease aqueous humor production or increase the outflow. Prostaglandin analogs, β-adrenoreceptor blockers, carbonic anhydrase inhibitors (CAIs), α_2_-adrenergic agonists, and Rho-associated coiled-coil containing protein kinase (Rho kinase, ROCK) inhibitors are the currently available IOP-lowering medications. As insufficient outcomes have been reported after the use of monotherapy treatments, a combination therapy has been subsequently used and found to lead to the successful management of glaucoma [3]. However, as compared to the administration of unfixed therapy, Barnebey et al. [4] previously reported that glaucoma patients exhibited both improvement and long-term adherence when using a fixed-combination therapy.

One novel IOP-lowering therapy is the use of ripasudil/brimonidine fixed-combination ophthalmic suspension (RBFC), for which there is the combined use of the Rho kinase inhibitor ripasudil and the α_2_-adrenergic agonist brimonidine. RBFC, which is the second type of fixed-combination IOP-lowering medication without β-adrenoreceptor blockers, has only been available in Japan since December 2022. Presently, prostaglandin analogues, β-adrenoreceptor blockers or CAIs are the fixed-dose combination that is commonly used to treat glaucoma. However, it is not recommended that a multiple fixed-dose combination with overlapping active ingredients be used to treat patients [5]. Therefore, as these common agents are not contained in RBFC, this can be used as a novel option for glaucoma medical treatment in combination with the existing IOP-lowering medications.

The present study investigated the efficacy and safety associated with the switch to RBFC from concomitant brimonidine and ripasudil, and brimonidine or ripasudil monotherapy, in glaucoma patients.

## 2. Materials and Methods

Between December 2022 and January 2024, data were retrospectively collected from all patients who were administered RBFC at Hiroshima University Hospital (Hiroshima, Japan), Hirota Eye Clinic (Yamaguchi, Japan), Kusatsu Eye Clinic (Hiroshima, Japan), Nagayama Eye Clinic (Okayama, Japan), Sagara Eye Clinic (Yamaguchi, Japan), Shirai Eye Hospital (Kagawa, Japan), and Suzuki Eye Clinic (Yamaguchi, Japan). This study protocol was approved by the Institutional Review Board of Hiroshima University (E2023-0173).

Best-corrected visual acuity (BCVA), refraction, fundus examination using indirect ophthalmoscopy, IOP, slit-lamp examination, gonioscopy examinations, and visual field test were performed in all patients. Patient identification, age, gender, glaucoma disease type, IOP, starting date of the RBFC administration, and current glaucoma therapy data that were recorded during the experiment were used for the analysis. IOPs, which were measured by a Goldmann applanation tonometer, were examined in the morning in all patients, with a single IOP measurement obtained at each visit. When patients chose to stop taking the medication or there were treatment regimen changes, these were all recorded where applicable. To be eligible for enrollment in the study, all subjects had to have an age ≥ 18 years, glaucomatous fundus abnormalities such as thinning of the rim in the optic disc or a defect in the retinal nerve fiber layer corresponding to visual field loss, and treatment with at least brimonidine 0.1% (Senju Pharmaceutical Co., Ltd., Osaka, Japan), or ripasudil (Kowa Co., Ltd., Aichi, Japan), or both brimonidine and ripasudil over 3 months. If subjects: (1) had a history of glaucoma surgery within one year of the RBFC administration or (2) were receiving systemic or local administration of a steroid during the follow-up period, they were excluded from the study. The eye having the higher IOP at the time of the baseline measurements was used for the analysis when both eyes met the inclusion criteria. The right eye was selected for the analysis if both eyes had the same IOP at baseline.

Patient examinations were performed at four visits that occurred over 24 weeks (day 0 and weeks 4, 12 and 24). If patients were treated by brimonidine or ripasudil monotherapy, or concomitant brimonidine and ripasudil, at day 0 (baseline), they were switched to RBFC. All other IOP-lowering medications were continued. IOP measurements by a Goldmann applanation tonometer in the morning, BCVA, fundus examination using indirect ophthalmoscopy, and slit-lamp examination were performed in all patients at each visit.

For conjunctival hyperemia assessment and to determine the hyperemia matching, a 4-point scale using a standard photograph was utilized. This scale included: 0 = no observed dilatation of vessels, 1 = dilatation of mainly small vessels, 2 = dilatation of small and large vessels and 3 = marked dilatation of small and large vessels [6]. Furthermore, superficial punctate keratopathy (SPK) was recorded using the slit-lamp microscope based on an A (area) D (density) grading scale [7].

For continuous variables, the variance equality was assessed through the use of the Anderson–Daring test. Differences between the baseline and every follow-up visit were assessed depending on the results obtained by either a Wilcoxon signed-rank test or Student’s *t*-test. All statistical analyses were performed using JMP software version 17 (SAS Inc., Cary, NC, USA). When the *p* value was 0.05 or less, this difference was defined as being significant. All statistical values are presented as the mean ± standard deviation.

## 3. Results

**Figure 1:**
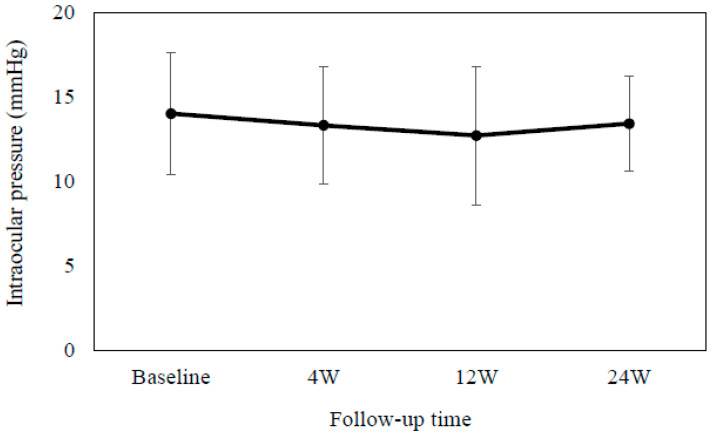
Mean intra-ocular pressure at baseline and at 4, 12 and 24 weeks after switching from concomitant brimonidine and ripasudil to RBFC. There was no significant difference observed in the mean intra-ocular pressure as compared to the baseline. Error bars show the standard deviation.

After switching 25 patients (11 men and 14 women, with a mean age of 71.2 ± 11.0 years) from concomitant brimonidine and ripasudil to RBFC, we first evaluated the associated efficacy and safety. Among these patients, 21 had POAG, 1 had exfoliation glaucoma, and 3 had uveitic glaucoma. Table 1 shows the patient characteristics. The mean IOP was as follows: 14.0 ± 3.6 mmHg at baseline, 13.3 ± 3.5 mmHg (*p* = 0.18) at 4 weeks, 12.7 ± 4.1 mmHg (*p* = 0.05) at 12 weeks, and 13.4 ± 2.8 mmHg (*p* = 0.93) at 24 weeks, with no significant change from baseline observed (Figure 1). As shown in Table 2, the AD grading scale or the conjunctival hyperemia scores were used to assess the degree of SPK or conjunctival hyperemia, respectively. At all of the evaluated time points, there were no significant differences. However, due to blepharitis (1 patient) and foreign body sensation (1 patient), the RBFC was discontinued in 2 patients.

**Figure 2:**
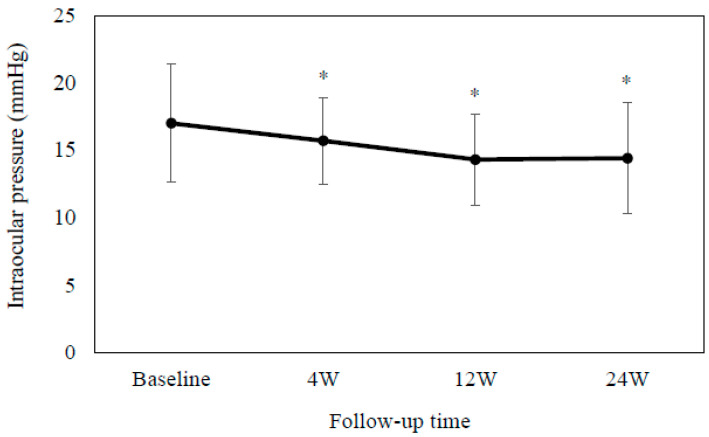
Mean intra-ocular pressure at baseline and at 4, 12 and 24 weeks after switching from brimonidine or ripasudil monotherapy to RBFC. There was a significant difference observed in the mean intra-ocular pressure as compared to the baseline. Error bars show the standard deviation. * *p* < 0.001.

**Figure 3:**
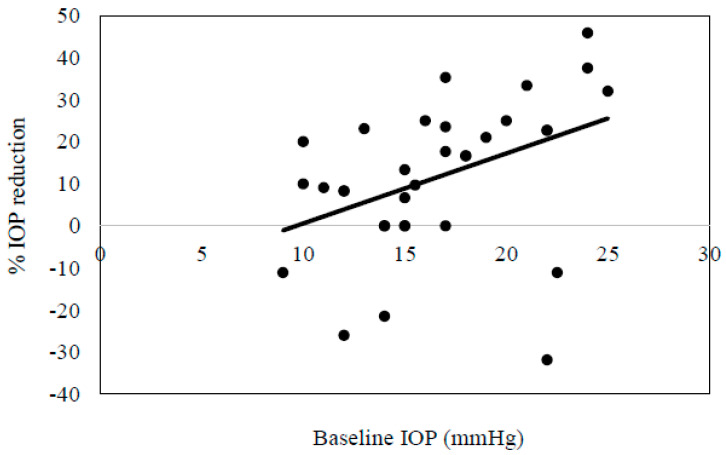
Scatter plot showing the correlation between baseline intra-ocular pressure and % intra-ocular pressure reduction at 6 months after switching from brimonidine or ripasudil to RBFC (*ρ* = 0.48, *p* = 0.004).

Second, after switching 45 patients (20 men and 25 women, with a mean age of 69.1 ± 9.8 years) from brimonidine (n = 40) or ripasudil (n = 5) monotherapy to RBFC, we also evaluated the associated efficacy and safety. Among these patients, 31 had POAG, 10 had exfoliation glaucoma, and 3 had uveitic glaucoma. Table 3 shows the patient demographic data. The mean IOP was as follows: 17.0 ± 4.4 mmHg at baseline, 15.7 ± 3.2 mmHg (*p* = 0.0003) at 4 weeks, 14.3 ± 3.4 mmHg (*p* < 0.0001) at 12 weeks, and 14.4 ± 4.1 mmHg (*p* = 0.005) at 24 weeks (Figure 2). Results between the baseline and each visit indicated that there were significant differences at each time point. Figure 3 shows the correlation between baseline IOP and % IOP reduction at 6 months after switching from brimonidine or ripasudil to RBFC. There was a positive correlation between the baseline IOP and % IOP reduction at 6 months after switching from brimonidine or ripasudil to RBFC (*ρ* = 0.48, *p* = 0.004). As shown in Table 4, there were no significant differences in the degree of the SPK or conjunctival hyperemia at any of the evaluated time points. RBFC was discontinued in four patients due to blepharitis (three patients) and itching (one patient).

## 4. Discussion

After switching to RBFC from concomitant brimonidine and ripasudil, IOP values were sustained throughout the 24-week observation period. However, after switching to RBFC from brimonidine or ripasudil monotherapy, there was a significant decrease in the IOP.

Inoue et al. [8] recently investigated changes in the IOP and found that, as compared to the baseline value of 15.1 ± 3.3 mmHg, at 3 months it was 15.9 ± 3.6 mmHg, while it was 14.6 ± 3.3 mmHg at 6 months after switching. However, these changes from baseline were not significant. In their study, the mean age was 65.5 years, with 42 men and 27 women, whereas in the present study, the mean age was 71.2 years, with more women than men evaluated. Although the background of the patients was somewhat different, our findings support the results of this previous study. In patients with primary open-angle glaucoma or ocular hypertension, the IOP-lowering effect of RBFC has been reported to be superior to that of brimonidine or ripasudil alone over 8 weeks [9]. In the group that was switched from ripasudil to RBFC, 47% were male and the mean age was 63.2 years, while in the group that was switched from brimonidine to RBFC, 53% were male and the mean age was 62.5 years [9]. Changes in the IOP after switching from ripasudil to RBFC were −2.6 mmHg [9]. Furthermore, changes in the IOP were −3.4 mmHg after switching from brimonidine to RBFC [9]. In the present study, after switching from brimonidine or ripasudil monotherapy to RBFC, we found the changes in the IOP to be −2.6 mmHg.

The preservative used in ripasudil and RBFC is 0.002% benzalkonium chloride (BAC). In contrast, the preservative in brimonidine does not contain BAC, but instead uses 0.005% sodium chlorite (NaClO_2_). A previous study found that there was toxicity on human corneal epithelial cell sheets associated with BAC concentrations that were higher than 0.01% [10]. After corneal epithelial cells underwent a 30-min exposure to 0.002–0.1% NaClO_2_, another study found that the survival rate was 80% or more [11]. The results from this study suggested that there is relatively little corneal epithelial damage after exposure to 0.002% BAC or 0.005% NaClO_2_. Therefore, after switching to RBFC, we found that there was no significant difference in the AD grading scale.

Blepharitis was the most commonly reported adverse event in the present study, with blepharitis also previously having been reported in patients treated with brimonidine [12,13] or ripasudil [14,15]. A significant risk factor for the onset of blepharitis following ripasudil administration is a history of an allergic reaction to other IOP-lowering medication [15,16]. In the present study, four eyes out of the 70 patients (5.7%) were found to have blepharitis, with most of these events reported to be mild in severity. In comparison, at 52 weeks, the incidence of blepharitis was found to be 17% [17]. Thus, a shorter observational period (24 weeks vs. 52 weeks) could be one possible explanation as to why the incidence of blepharitis was lower. In fact, the highest blepharitis incidence occurred between weeks 24 and 36 [17].

Glaucoma treatments using multi-drug regimens often result in adherence issues due to the inconvenience of the treatments. Therefore, there are some merits to switching to fixed-dose combination therapies. In addition, it has been reported that there was improved convenience and patient satisfaction after changing from brinzolamide and brimonidine to a brinzolamide/brimonidine fixed-combination ophthalmic suspension (BBFC), due to the decrease in the number of IOP-lowering medications [18]. Furthermore, patients were more aware of the effectiveness of the treatment after switching from brinzolamide or brimonidine to BBFC, because of the decrease in the IOP [19]. Thus, there should be an improved adherence due to a reduction in the medication burden after implementing fixed-dose combination therapies.

Several patients had increased IOP after switching to RBFC (Figure 3). These patients previously had significant IOP fluctuations, and the increase observed in the present study is considered to be within the range of typical IOP fluctuations. Two of these patients subsequently underwent trabeculectomy.

The present study had some limitations. First, only a short observation period was utilized in this study. In order to obtain clearer, more definitive results, a longer observation period will be required. Second, this study only analyzed a small number of samples and was a retrospective study. Thus, large-scale and prospective studies will need to be conducted in the future to obtain more rigorous and definitive evidence. Third, after the change to RBFC, other IOP-lowering medications were also continued. Therefore, the possibility that the use of other IOP-lowering medications may have affected the change in IOP after switching to RBFC cannot be ruled out. Fourth, 40 patients were treated with brimonidine and five patients were treated with ripasudil before switching to RBFC. This discrepancy in sample sizes between the brimonidine and ripasudil groups suggests that the observed results are predominantly influenced by the effects of the brimonidine treatment.

## 5. Conclusions

Sustained IOP values were obtained after switching from concomitant brimonidine and ripasudil to RBFC throughout a 24-week evaluation period. In contrast, switching to RBFC from brimonidine or ripasudil monotherapy resulted in a significant decrease in the IOP. Furthermore, the present results show not only that the use of RBFC was safe, but also that there was a low incidence of adverse drug reactions or treatment discontinuation in these patients.

## Figures and Tables

**Table 1:** Baseline characteristics of patients treated with concomitant.

Brimonidine and Ripasudil	
Age (years)	71.2 ± 11.0
Gender (M/F)	11/14
Baseline IOP (mmHg)	14.0 ± 3.6
Type of glaucoma	
POAG	21
Exfoliation glaucoma	1
Uveitic glaucoma	3
IOP-lowering medications	
(except brimonidine and ripasudil)	
PGA	13
PGA/β-blocker	6
CAI	2
β-blocker/CAI	2
PGA+β-blocker/CAI	2

M: male, F: female, IOP: intra-ocular pressure, POAG: primary open-angle glaucoma, PGA: prostaglandin analogue, CAI: carbonic anhydrase inhibitor.

**Table 2:** SPK and hyperemia scores.

	SPK Score	*p* Value *	Hyperemia Score	*p* Value *
Baseline	1.38 ± 1.19		0.53 ± 0.64	
4W	0.89 ± 1.45	0.38	0.60 ± 0.84	0.63
12W	1.27 ± 1.01	0.28	0.46 ± 0.66	0.63
24W	1.43 ± 0.98	>0.99	0.43 ± 0.55	>0.99

* Calculated using Wilcoxon signed-rank test. SPK: superficial punctate keratopathy.

**Table 3:** Baseline characteristics of patients who were treated with brimonidine or ripasudil monotherapy.

Age (years)	69.1 ± 9.8
Gender (M/F)	20/25
Baseline IOP (mmHg)	17.0 ± 4.4
Type of glaucoma	
POAG	32
Exfoliation glaucoma	10
Uveitic glaucoma	3
IOP-lowering medications	
(except brimonidine and ripasudil)	
PGA	16
PGA/β-blocker	13
β-blocker/CAI	8
PGA+β-blocker/CAI	2
none	6

M: male, F: female, IOP: intra-ocular pressure, POAG: primary open-angle glaucoma, PGA: prostaglandin analogue, CAI: carbonic anhydrase inhibitor.

**Table 4:** SPK and hyperemia scores.

	SPK Score	*p* Value *	Hyperemia Score	*p* Value *
Baseline	0.63 ± 1.13		0.39 ± 0.58	
4W	0.50 ± 1.04	0.07	0.57 ± 0.79	0.10
12W	0.57 ± 1.07	0.68	0.45 ± 0.65	0.53
24W	0.61 ± 0.90	0.73	0.39 ± 0.56	0.53

* Calculated using Wilcoxon signed-rank test. SPK: superficial punctate keratopathy.

## Data Availability

The datasets and materials used and/or analyzed during the current study are available from the corresponding author upon reasonable request.
